# Diagnostic Approach to Biliary Strictures

**DOI:** 10.3390/diagnostics15030325

**Published:** 2025-01-30

**Authors:** Daniyal Raza, Sahib Singh, Stefano Francesco Crinò, Ivo Boskoski, Cristiano Spada, Lorenzo Fuccio, Jayanta Samanta, Jahnvi Dhar, Marco Spadaccini, Paraskevas Gkolfakis, Marcello Fabio Maida, Jorge Machicado, Marcello Spampinato, Antonio Facciorusso

**Affiliations:** 1 Department of Internal Medicine, LSU Health Shreveport, Shreveport, LA 71103, USA; daniyal.raza92@gmail.com; 2 Department of Internal Medicine, Sinai Hospital, Baltimore, MD 21215, USA; sahibs559@gmail.com; 3 Gastroenterology and Digestive Endoscopy Unit, University Hospital of Verona, 37134 Verona, Italy; stefanofrancesco.crino@aovr.veneto.it; 4 Digestive Endoscopy Unit, Fondazione Policlinico Universitario Agostino Gemelli IRCCS, 00136 Roma, Italy; ivo.boskoski@policlinicogemelli.it (I.B.); cristiano.spada@unicatt.it (C.S.); 5 Department of Medical Sciences and Surgery, University of Bologna, 40126 Bologna, Italy; lorenzofuccio@gmail.com; 6 Gastroenterology Unit, Post Graduate Institute of Medical Education and Research, Chandigarh 160012, India; dj_samanta@yahoo.co.in (J.S.); jahnvi3012@gmail.com (J.D.); 7 IRCCS Humanitas Research Hospital, Via Manzoni 56, Rozzano, 20089 Milano, Italy; marco.spadaccini@humanitas.it; 8 Department of Gastroenterology, “Konstantopoulio-Patision” General Hospital of Nea Ionia, 142 33 Athens, Greece; 9 Gastroenterology Unit, Umberto I Hospital Enna, 94100 Enna, Italy; marcello.maida@unikore.it; 10 Division of Gastroenterology and Hepatology, University of Michigan, Ann Arbor, MI 48109, USA; machicad@med.umich.edu; 11 General Surgery Unit, Vito Fazzi Hospital, 73100 Lecce, Italy; marcello.spampinato@gmail.com; 12 Gastroenterology Unit, Department of Experimental Medicine, University of Salento, 73100 Lecce, Italy

**Keywords:** biliary strictures, ERCP, EUS, MRI/MRCP, artificial intelligence, cholangioscopy, CECT, tumor markers

## Abstract

Biliary strictures represent a narrowing of the bile ducts, leading to obstruction that may result from benign or malignant etiologies. Accurate diagnosis is crucial but challenging due to overlapping features between benign and malignant strictures. This review presents a comprehensive diagnostic approach that integrates biochemical markers, imaging modalities, and advanced endoscopic techniques to distinguish between these causes. Imaging tools such as ultrasound, MRI/MRCP, and CECT are commonly used, each with distinct advantages and limitations. Furthermore, endoscopic procedures such as ERCP and EUS are key in tissue acquisition, enhancing diagnostic accuracy, especially for indeterminate or complex strictures. Recent innovations, including artificial intelligence and new endoscopic techniques, hold promise in enhancing precision and reducing diagnostic challenges. This review emphasizes a multidisciplinary strategy to improve diagnostic pathways, ensuring timely management for patients with biliary strictures.

## 1. Introduction

A biliary stricture refers to a constriction within the liver’s bile ducts that can obstruct bile flow and lead to significant clinical and physiological effects. This blockage often results in upstream ductal dilation, producing both pathological changes and symptoms associated with biliary obstruction. While some patients remain asymptomatic, with strictures incidentally identified on imaging, others may present with symptoms such as pruritus or jaundice [1].

Biliary strictures may result from both benign and malignant causes. Malignancies—either primary or metastatic—are common and have critical implications for diagnosis and treatment. For patients with biliary strictures without a discernible mass on cross-sectional imaging, the risk of malignancy is approximately 55% [2]. Benign causes include conditions such as primary sclerosing cholangitis, IgG4-related sclerosing cholangitis, fibrotic strictures, and chronic pancreatitis. Benign strictures usually present with tapered margins and smooth, symmetric borders, while malignant strictures are marked by shouldered margins and irregular, asymmetric borders [3]. Malignant strictures also typically involve longer segments compared to the shorter segments seen in benign strictures and show enhancement on contrast-enhanced cross-sectional imaging [4].

However, benign strictures can appear similar to malignant ones on imaging, necessitating tissue acquisition to accurately differentiate between the two [5]. Diagnosing biliary strictures thus requires a comprehensive approach, integrating biochemical markers, imaging techniques like CT and MRI, and specialized endoscopic methods for direct visualization and tissue sampling.

In this review, we will examine current evidence and guidelines for the diagnostic approach to biliary strictures, with a focus on distinguishing benign from malignant cases through a multidisciplinary evaluation.

## 2. Prevalence and Etiology

The overall annual cost of biliary diseases in the United States, including conditions like gallbladder disease, choledocholithiasis, and other non-obstructive biliary disorders, is estimated to be around USD 16.9 billion. However, accurately determining the prevalence of biliary strictures remains difficult due to the lack of a specific administrative coding system [6]. Bile duct strictures, which may be congenital or more commonly acquired, often pose diagnostic and therapeutic challenges [7]. Acquired strictures can be benign or malignant, with benign causes representing about 30% of cases [1]. Iatrogenic injury during laparoscopic cholecystectomy is a leading cause of benign strictures, while malignant strictures are most frequently associated with cancers of the pancreatic head and bile ducts, such as cholangiocarcinoma [8,9]. Each year, approximately 57,000 new pancreatic cancer cases are diagnosed, with an estimated 60% causing obstructive jaundice and contributing to malignant biliary strictures [8]. In some cases, strictures remain indeterminate, defined as those without an associated mass on imaging and undetermined as benign or malignant even after endoscopic retrograde cholangiopancreatography (ERCP) with sampling [10]. Given the high prevalence of malignancy in biliary strictures, these indeterminate cases warrant careful monitoring for any features suggestive of a malignant origin.

Accurate diagnosis is crucial but often complex, requiring a multidisciplinary approach involving biochemical testing, imaging, and advanced endoscopic techniques to differentiate between benign and malignant etiologies. Table 1 summarizes the diverse causes of biliary strictures, highlighting the need for comprehensive diagnostic protocols to guide effective management and improve patient outcomes.

## 3. Laboratory Tests

The initial assessment of biliary strictures should start with a noninvasive approach, incorporating history, physical examination, and laboratory tests. Understanding the patient’s surgical history, particularly prior procedures like cholecystectomy, is essential, as these can lead to biliary or anastomotic strictures. Laboratory tests are conducted after obtaining the patient’s history and evaluating their symptoms.

Carbohydrate antigen 19-9 (CA 19-9) and carcinoembryonic antigen (CEA) are the primary tumor markers studied for detecting malignancies in the pancreato-biliary system. These glycoprotein markers have proven utility in assessing prognosis, recurrence, staging, and surgical resectability, as demonstrated in various studies [11,12,13,14]. However, their effectiveness in distinguishing malignant from benign biliary obstructions, especially in jaundiced patients, is controversial due to high rates of false positives.

Although CA 19-9 is frequently elevated in malignancies like pancreatic, cholangiocarcinoma, and gallbladder cancers, its diagnostic accuracy is compromised in patients with cholestasis or cholangitis, where sensitivity and specificity can be as low as 74% and 41.5%, respectively [15]. Moreover, CA 19-9 levels can also rise in benign conditions, including primary sclerosing cholangitis, cirrhosis, and obstructive jaundice [16]. Similarly, CEA levels can be elevated in other malignancies, such as colorectal, gastric, and breast cancers, and lack strong correlation with pancreato-biliary conditions specifically [17]. A meta-analysis found that CA 19-9 had a pooled diagnostic accuracy of 81%, with sensitivities and specificities ranging widely [17,18]. However, due to significant heterogeneity among studies, the reliability of these findings is limited.

When CA 19-9 and CEA were combined with imaging, sensitivity and specificity improved, underscoring that tumor markers alone are insufficient for accurate diagnosis. For instance, Morris-Stiff et al. showed that using CA 19-9 alongside imaging increased sensitivity and specificity from 84.9% to 97.2% and from 69.7% to 88.7%, respectively [19]. Table 2 summarizes the tumor markers CA 19-9 and CEA used for assessing malignancy in biliary strictures, highlighting their sensitivity, specificity, diagnostic utility, and limitations, including their lack of specificity in differentiating between benign and malignant conditions.

In summary, while tumor markers such as CA 19-9 and CEA may aid in diagnosis, they lack the specificity to reliably differentiate between benign and malignant biliary strictures when used in isolation, particularly in jaundiced patients. These markers should be used as part of a broader diagnostic approach, including imaging and histopathological evaluation, to ensure a more accurate diagnosis.

## 4. Cross-Sectional Imaging

In patients presenting with jaundice or laboratory evidence of cholestasis and suspected biliary strictures, the diagnostic approach typically begins with clinical and laboratory evaluations, followed by imaging studies. Trans-abdominal ultrasonography is often the first imaging choice due to its accessibility, cost-effectiveness, and ability to quickly detect bile duct dilation. However, its sensitivity and specificity vary widely (31–100% and 71–97%, respectively), limiting its accuracy in precisely locating the stricture or distinguishing between benign and malignant causes [20,21,22].

Positron Emission Tomography-Computed Tomography (PET-CT) with 18F-FDG offers an additional imaging modality for evaluating biliary strictures, particularly in staging biliary tract cancers (BTCs). It provides high sensitivity (up to 90.1%) and specificity (83.5%) for detecting relapse and metastases. Moreover, PET-CT has been shown to alter management in approximately 15% of cases by identifying previously undetected disease sites. However, its specificity for differentiating benign from malignant strictures remains low, underscoring the need for histological confirmation in equivocal cases [23].

Given these limitations, magnetic resonance imaging with magnetic resonance cholangiopancreatography (MRI/MRCP) and contrast-enhanced computed tomography (CECT) have emerged as primary imaging modalities for comprehensive assessment of biliary strictures. MRI/MRCP, using T2-weighted sequences, provides high-contrast visualization of bile-filled structures against surrounding tissues [24]. This non-invasive approach avoids radiation and contrast agents, offering multi-planar views that help locate, evaluate, and measure obstructions. For assessing obstructive jaundice, MRCP’s sensitivity, specificity, and accuracy range from 81 to 100%, 84 to 100%, and 90 to 96%, respectively [25]. Additionally, studies show MRI/MRCP’s diagnostic accuracy surpasses that of CECT for determining both the stricture’s location (OR 3.31, 1.20–9.09) and malignancy (96% vs. 89%; OR 2.07, 1.18–3.03) [25,26].

While CECT is advantageous for its speed and reduced motion artifacts, it involves radiation exposure and requires intravenous contrast, making it less suitable in certain cases. MRI/MRCP also reduces the need for unnecessary endoscopic retrograde cholangiopancreatography (ERCP) in about one-third of cases [27] and serves as an alternative when ERCP is unsuccessful or contraindicated, such as in pregnant patients with suspected biliary obstruction [28]. MRI/MRCP is particularly valuable for planning therapeutic ERCP in complex cases, including hilar strictures and instances requiring concurrent pancreatic interventions [27]. Table 3 illustrates different imaging modalities used in the diagnosis of biliary strictures, including their respective sensitivities, advantages, and limitations.

In conclusion, MRI/MRCP is preferred over CECT for evaluating biliary strictures in patients with suspected obstruction, as it provides superior accuracy in identifying the level and nature of the stricture and aids in comprehensive treatment planning.

## 5. Endoscopic Evaluation

### 5.1. Extrahepatic Strictures

**Figure 1:**
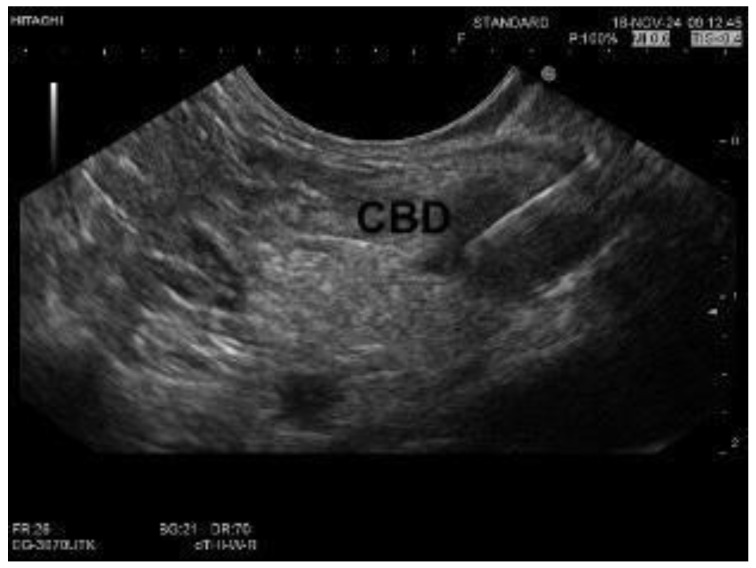
EUS-FNB of an extra-hepatic biliary stricture. CBD, common bile duct.

The evaluation of biliary strictures primarily involves two main techniques: ERCP-based tissue sampling, including brush cytology and forceps biopsies, and EUS-guided fine-needle aspiration or biopsy (EUS-FNA/B; Figure 1). A recent randomized controlled trial assessed the sensitivity of two intraductal brush cytology devices during ERCP in patients with suspected malignant extrahepatic biliary strictures. The study, which included 64 patients randomized to either a dense or conventional brush, found the sensitivity of the dense brush (50%) was not significantly superior to the conventional brush (44%). This underscores the inherent challenges of achieving high sensitivity with current ERCP-based cytological methods and highlights the need for complementary diagnostic tools [29].

Traditionally, ERCP-based techniques such as brush cytology and intraductal biopsies have been prevalent, though their sensitivity for detecting malignancy is often limited when used in isolation. A recent multicenter randomized trial comparing the diagnostic sensitivity and cellular yield of two intraductal brush cytology devices found that the aggressive Infinity brush achieved a sensitivity of 79% compared to 67% for the standard RX Cytology Brush, though the difference was not statistically significant (*p* = 0.10). However, the Infinity brush provided a significantly higher cellular yield (*p* < 0.001), indicating that improvements in brush design may not markedly enhance diagnostic sensitivity [30].

A systematic review and meta-analysis analyzing 16 trials provided further insights into optimizing ERCP techniques. This study found that increasing the number of brush passes and sending bile fluid for cytology could enhance the sensitivity of biliary brushings by up to 20% and 16%, respectively. However, interventions such as stricture dilation before brushing and alternative brush designs did not significantly improve sensitivity [31]. In addition, a randomized controlled trial by Shang et al. (2024) demonstrated that modified pediatric gastroscopy biopsy forceps improved diagnostic sensitivity and accuracy during ERCP procedures for biliary strictures. The modified forceps, featuring a bent tip for better biliary access, achieved an 83.3% sensitivity compared to 50.0% with conventional forceps, significantly enhancing diagnostic outcomes without increasing complication rates [32]. These findings suggest that innovative biopsy instrument modifications can address limitations of traditional methods and provide more reliable diagnostic results.

Meta-analyses indicate that adding intraductal biopsies to ERCP with brush cytology improves sensitivity by about 20%, resulting in a combined sensitivity of 66%, compared to 40% for brush cytology alone [5]. EUS-TA has shown even greater sensitivity in diagnosing distal strictures, with a pooled sensitivity of 83% [33].

A 2024 prospective study by Park et al. evaluated the utility of massive parallel sequencing (MPS) on biliary brush cytology and bile fluid samples obtained via ERCP for diagnosing malignant bile duct strictures [5]. The use of MPS identified key mutations (e.g., TP53, BRAF, CTNNB1) associated with extrahepatic cholangiocarcinoma, indicating that MPS-based molecular assays could be integrated into current diagnostic protocols to enhance early detection and guide targeted treatment strategies.

Fluorescence in situ hybridization (FISH) has emerged as an important molecular tool, identifying chromosomal abnormalities through fluorescent DNA probes targeting loci such as chromosomes 3, 7, and 17, and the 9p21 locus (P16) [34]. Combining FISH with brush cytology enhances diagnostic sensitivity, increasing it from 35–52% to up to 84.2%, albeit with a specificity of 54.1% [34,35,36]. Despite its advantages, FISH can be labor-intensive and subject to variability due to nuclear overlap, affecting probe interpretation.

**Figure 2:**
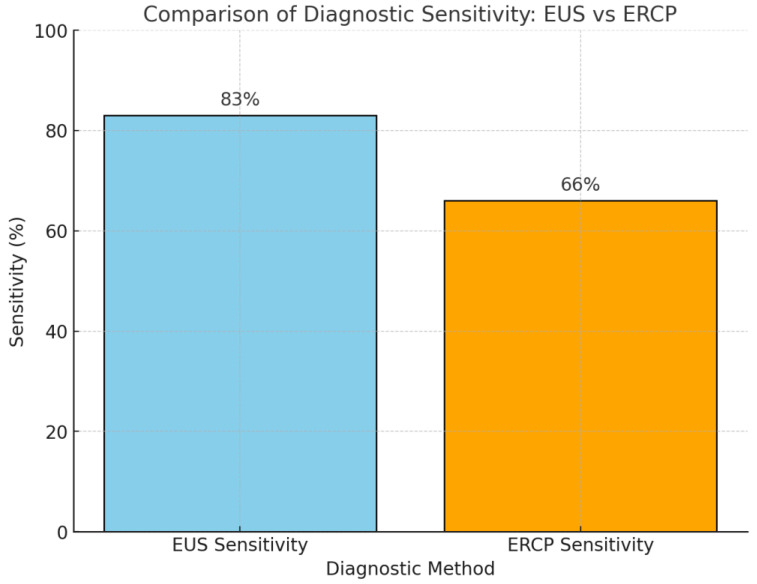
Comparison of sensitivity between EUS-TA and ERCP for diagnosing distal biliary strictures [32].

The AJG guidelines recommend endoscopic ultrasound (EUS) with fine-needle aspiration or biopsy (FNA/B) as the preferred diagnostic approach when an extrahepatic biliary stricture is associated with a suspected or apparent pancreatic mass, instead of relying solely on ERCP. This strong recommendation is supported by moderate-quality evidence [20]. Additionally, in cases of incidental common bile duct (CBD) dilation without abnormal lab results, EUS remains a highly valuable diagnostic tool. Despite advances in endoscopic technology, earlier studies have consistently shown that EUS-FNA can be more effective than ERCP-based approaches, particularly in cases of suspected malignancy. For example, a 2004 prospective study involving patients with obstructive jaundice demonstrated that EUS-guided sampling achieved a sensitivity of 60%, surpassing ERCP’s 38% sensitivity for suspected pancreatic masses [26]. More recent studies corroborate EUS-FNA’s effectiveness; a 2014 prospective study involving 51 patients reported superior diagnostic sensitivity and accuracy of EUS-FNA over ERCP-based tissue sampling, particularly for pancreatic malignancies, with EUS-FNA achieving a sensitivity of 94% versus 50% for ERCP [37]. High-resolution imaging methods such as contrast-enhanced computed tomography (CECT) and MRI/MRCP have increased the detection of incidental CBD dilation. EUS can uncover significant findings, including CBD stones and, in rare instances, malignancies, even in patients with normal laboratory results [38]. This comprehensive approach reduces the risk of missed diagnoses. EUS-TA has demonstrated high diagnostic accuracy, with one study reporting a sensitivity of 94% for suspected malignant biliary strictures compared to 50% and 53% for ERCP-based cytology and biopsies, respectively [37]. Figure 2 compares sensitivity of EUS and ERCP.

EUS-TA has shown some benefits in perihilar strictures. For instance, a study involving 44 patients with prior negative brush cytology found that EUS-FNA achieved a diagnostic accuracy of 91% and sensitivity of 89%, influencing surgical planning in nearly two-thirds of cases without complications [39]. However, the diagnostic performance of EUS-TA can vary between perihilar and distal strictures. A 2020 study involving 97 cases of biliary strictures (46% hilar) reported an overall EUS-FNA sensitivity of 75%. The highest sensitivity and negative predictive value (NPV) were observed in distal lesions without stents (95% and 93%, respectively), whereas perihilar lesions with stents showed lower sensitivity (56%) and NPV (33%) [39]. This underscores that EUS-TA tends to be less effective for perihilar strictures compared to distal ones.

Factors such as proximal location, presence of primary sclerosing cholangitis (PSC), and stenting were identified as independent factors that reduced the accuracy of EUS-TA in perihilar strictures. The diffuse growth patterns and unique biological characteristics of perihilar cholangiocarcinoma contribute to these lower detection rates compared to distal lesions [40,41].

EUS-TA also carries a risk of peritoneal seeding, potentially leading to tumor upstaging, which is generally a contraindication for patients eligible for liver transplantation. Although the risk is relatively low, a Japanese study reported a 0.33% incidence of needle tract seeding, primarily in cases of pancreatic adenocarcinoma [42]. This rate was notably higher compared to patients who underwent intraductal sampling or did not receive a biopsy. A notable study conducted by the Mayo Clinic found peritoneal metastasis in 83% of patients (five out of six) who had a positive transperitoneal biopsy—either endoscopic or percutaneous—for cholangiocarcinoma during liver transplant evaluation [43]. Evidence suggests that EUS-FNA poses a lower risk of seeding compared to percutaneous approaches [44]. This risk should be balanced against the benefits, especially in patients with non-resectable disease where EUS-TA may be more readily considered. Notably, EUS-FNA/B or percutaneous biopsy often provides higher diagnostic accuracy than ERCP-based sampling when evaluating a hilar mass or bile duct thickening [45]. However, the potential risk of transperitoneal needle tracking, which may lead to peritoneal seeding, remains a concern. The combination of brush cytology and fluoroscopy-guided biopsies during ERCP remains an effective approach for tissue diagnosis in proximal biliary strictures. EUS-TA proves particularly useful when ERCP-based methods yield inconclusive results. A meta-analysis found that incorporating EUS after an inconclusive ERCP with brushing increased the diagnostic yield by 15% (95% CI: 9–24%) [45]. In cases where cross-sectional imaging shows accessible extraluminal disease, EUS-TA offers a minimally invasive sampling option, with pooled sensitivity and accuracy of 78% and 84%, respectively [46,47].

For patients with extrahepatic biliary strictures linked to suspected or apparent pancreatic masses, the AJG recommends performing EUS and ERCP in a single session, which facilitates simultaneous diagnosis and drainage. This combined approach enhances diagnostic yield, achieving accuracy rates between 92.5% and 98%, significantly higher than using either method alone [46,47,48].

Single-session EUS and ERCP offer additional advantages, including procedural efficiency and patient convenience, without increasing the risk of complications. A randomized controlled trial involving 180 patients with obstructive jaundice found no significant differences in procedural time, anesthesia requirements, or complication rates between single-session and separate-session approaches [49]. This approach reduces the need for repeated anesthesia and enhances overall efficiency. In some cases, EUS can provide sufficient anatomical information, potentially eliminating the need for ERCP when EUS findings clarify the diagnosis [50].

Advancements in EUS fine-needle biopsy (EUS-FNB) with end-cutting biopsy needles have further improved diagnostic accuracy by enhancing tissue yield, particularly in cases involving small lesions or thickened bile ducts [50]. EUS-FNB offers greater reliability compared to traditional EUS-FNA, establishing itself as a valuable tool for evaluating malignancy in distal biliary strictures.

Beyond diagnosis, EUS enables comprehensive staging of malignancies, assessment of lymph node involvement, and detection of potential liver metastases, which are essential for treatment planning and prognosis [51]. This capability makes EUS a critical component of a multimodal approach to distal biliary stricture evaluation, supporting more informed therapeutic decisions.

While EUS-TA is highly effective, it poses potential risks, such as bile leakage or cholangitis, especially in cases involving a non-drained biliary system or narrowed distal bile ducts [52]. This underscores the importance of a tailored approach, considering individual patient anatomy and disease characteristics during diagnostic and therapeutic planning.

Notably, the studies supporting this combined diagnostic strategy are primarily observational or retrospective, leading to a very low-quality evidence rating per GRADE criteria [49,53]. Despite this, the consistent and significant findings across studies justify incorporating EUS and ERCP for more accurate diagnosis and comprehensive assessment of distal biliary strictures.

Combining EUS-TA with ERCP-based sampling enhances diagnostic sensitivity and minimizes non-diagnostic outcomes, particularly in patients presenting with jaundice. The single-session EUS and ERCP approach improves procedural efficiency without increasing adverse events, while advancements in EUS-FNB enhance tissue yield and diagnostic accuracy. Collectively, these techniques offer a robust framework for managing distal biliary strictures, enabling early and accurate diagnosis, staging, and tailored treatment planning.

**Figure 3:**
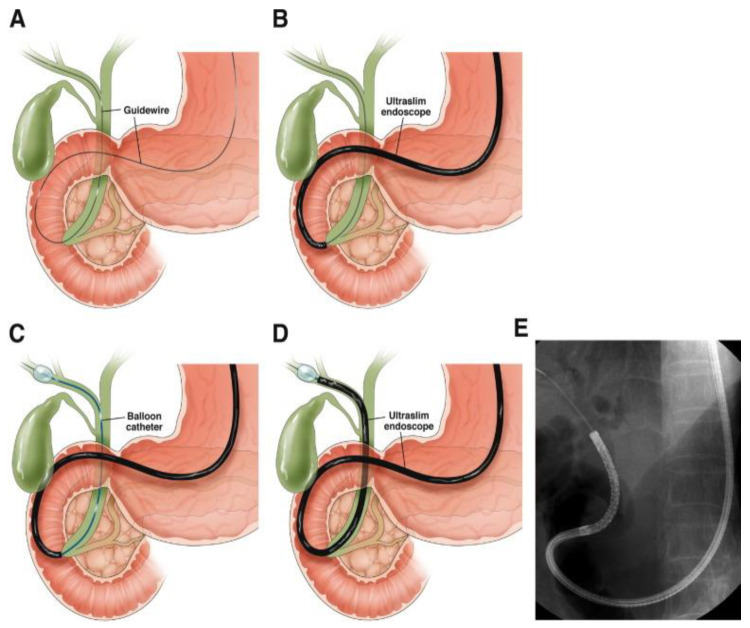
This sequence of images illustrates the procedural steps of intraductal balloon-guided direct peroral cholangioscopy. (**A**): Placement of a guidewire, with or without a 5-French drainage catheter, into a branch of the intrahepatic duct for securing access. (**B**): Introduction of an ultraslim endoscope, guided along the wire, to the ampulla of Vater. (**C**): Insertion and inflation of a 5-French balloon catheter to anchor within the duct, ensuring stability during endoscopic advancement. (**D**): Advancement of the ultraslim endoscope over the fixed balloon catheter to enable direct visualization of the biliary tract. (**E**): Radiographic confirmation of the ultraslim endoscope’s placement within the proximal biliary tree, allowing for precise diagnostic and therapeutic maneuvers [76].

A 2024 retrospective study by Guillén-Graf et al. highlighted the utility of digital single-operator cholangioscopy (DSOC) for evaluating biliary strictures post-orthotopic liver transplantation (OLT) [54]. Reviewing post-OLT patients from 2019 to 2022, the study reported a 27% incidence of biliary complications within one to eight months. DSOC provided high-quality imaging, aiding in the precise diagnosis and management of complex strictures that conventional ERCP might miss. It also facilitated endoscopic interventions like stenting and dilation, improving treatment outcomes. This underscores the value of integrating DSOC into post-OLT management protocols. Additionally, DSOC has also demonstrated significant safety and diagnostic accuracy in patients awaiting orthotopic liver transplantation (OLT). Studies indicate a low complication rate comparable to other diagnostic modalities, reinforcing its role as a safe option in this high-risk population [55]. Furthermore, DSOC’s ability to provide real-time visualization and targeted biopsies enhances diagnostic confidence, particularly in identifying and characterizing biliary strictures prior to orthotopic liver transplantation. By facilitating precise diagnosis, DSOC can influence transplant candidacy and surgical planning, underscoring its clinical value in the pre-transplant evaluation process. Figure 3 shows the sequence of images illustrates the steps involved in intraductal balloon-guided direct peroral cholangioscopy.

Recent progress in diagnosing malignant biliary strictures (MBS) has emphasized the role of molecular techniques, including methylated DNA markers (MDMs). A study by Cooley et al. (2024) evaluated the utility of MDMs from biliary brushings in detecting MBS in a prospective cohort. The study found that four key MDMs—TWIST1, HOXA1, VSTM2B, and CLEC11A—demonstrated strong diagnostic performance, with individual AUC values ranging from 0.78 to 0.83 and high specificity (95.2–95.3%). When combined, the MDM panel achieved an AUC of 0.86, with a sensitivity of 73.4% and specificity of 92.9%, surpassing traditional methods like cytology and FISH [56].

The diagnostic evaluation of extrahepatic biliary strictures faces notable limitations despite advancements in techniques. Traditional ERCP-based tissue sampling methods, including brush cytology and forceps biopsy, continue to exhibit low sensitivity and specificity, with malignancy detection rates ranging between 40 and 66% [5]. These limitations contribute to false negatives, resulting in delayed diagnosis and suboptimal treatment initiation. While advanced techniques such as EUS-guided fine-needle aspiration (EUS-FNA) and intraductal ultrasound (IDUS) provide higher diagnostic yields, their invasive nature and reliance on specialized training create barriers to widespread implementation. Additionally, complications such as bile leakage and cholangitis are significant concerns, especially in patients with undrained biliary systems or thickened ducts [52]. High costs and the need for specialized expertise further limit the availability of advanced diagnostic modalities such as IDUS and confocal laser endomicroscopy (CLE), particularly in resource-constrained settings [57,58].

Future advancements in the diagnostic approach to extrahepatic biliary strictures offer significant promise. Enhancing EUS-guided fine-needle biopsy (EUS-FNB) with next-generation needle designs and innovative tissue acquisition strategies could significantly improve the accuracy and reliability of diagnoses. Artificial intelligence (AI), particularly in the form of convolutional neural networks (CNNs), holds the potential to revolutionize diagnostic imaging by reducing inter-observer variability and providing real-time insights during procedures like cholangioscopy and EUS [59]. Furthermore, personalized medicine approaches, including genomic profiling and targeted therapies, are anticipated to refine diagnostic and treatment strategies for extrahepatic biliary strictures, ensuring more tailored and effective management plans [60,61,62,63,64].

### 5.2. Perihilar Structures

Diagnosing and managing perihilar biliary strictures continues to pose significant challenges due to overlapping imaging characteristics between benign and malignant cases. Historical data indicate that approximately 15% of patients undergoing surgery for presumed perihilar cholangiocarcinoma (PHC) are ultimately found to have benign strictures, a phenomenon known as the “malignant masquerade” [65]. While imaging features such as lobar atrophy, soft tissue mass, and vascular involvement are more suggestive of malignancy, these characteristics can also be present in benign conditions, complicating preoperative evaluations. Standard diagnostic methods, including bile duct brushings and biopsy, often yield suboptimal sensitivity and specificity (82% and 55%, respectively), highlighting the need for improved diagnostic tools [65].

Accurate tissue diagnosis is crucial for patients with perihilar biliary strictures, as a significant portion of these cases are malignant, frequently associated with cholangiocarcinoma. To enhance diagnostic yield, the combination of brush cytology with fluoroscopy-guided biopsies during ERCP is often recommended. A meta-analysis of 21 observational studies demonstrated that adding fluoroscopy-guided biopsies to brush cytology increased diagnostic sensitivity by approximately 20%, achieving a combined sensitivity of 66% compared to 40% with brush cytology alone [5]. This combination is particularly effective for detecting malignancies in perihilar strictures, where diagnostic accuracy has historically been challenging.

Further studies emphasize the value of this combined approach in complex proximal strictures. For example, a cohort study involving 58 patients with hilar cholangiocarcinoma reported sensitivities of 41.4% for brush cytology, 53.4% for fluoroscopy-guided biopsies, and 60.3% when both methods were used together [47,56]. Another study using specialized double-balloon enteroscopy forceps on 43 patients with proximal strictures showed sensitivities of 49% for brushing, 69% for fluoroscopy-guided biopsies, and 80% for the combination [66]. Although these methods improve diagnostic yield, fluoroscopy-guided biopsies require advanced technical expertise and carry some risk of complications, such as bleeding and perforation. This necessitates a multidisciplinary approach to determine the most appropriate diagnostic strategy.

In summary, for patients with perihilar strictures, combining brush cytology with fluoroscopy-guided biopsies during ERCP enhances diagnostic accuracy. When ERCP-based methods are insufficient, EUS-TA serves as a valuable alternative, particularly in non-resectable cases or where extraluminal disease is accessible. This multimodal approach maximizes the likelihood of obtaining an accurate tissue diagnosis, supporting appropriate treatment planning in complex cases.

The diagnostic challenges associated with perihilar biliary strictures are compounded by the limitations of existing imaging and sampling modalities. While MRI/MRCP is instrumental in visualizing bile ducts, its diagnostic accuracy is often hindered by operator skill and imaging resolution, leading to missed subtle signs of malignancy in complex perihilar strictures [25,26]. Additionally, the diagnostic performance of EUS is notably reduced in perihilar strictures, particularly in patients with conditions such as primary sclerosing cholangitis (PSC) or those with indwelling stents [40]. A significant portion of the available evidence supporting these diagnostic approaches is derived from observational and retrospective studies, leading to variability in findings and contributing to a low-quality evidence base [50].

Looking ahead, advancements in MRI/MRCP, particularly the use of enhanced contrast agents and optimized imaging protocols, may improve the detection of perihilar strictures and reduce reliance on more invasive procedures. Endoscopic innovations, such as sheath-assisted tools for transpapillary biopsies, have demonstrated increased sample collection rates and higher sensitivity, offering a promising approach to diagnosing perihilar biliary strictures [59]. Furthermore, genomic analysis combined with tissue sampling has the potential to distinguish malignant from benign strictures with greater precision [60,61,62,63,64]. These advancements are expected to significantly improve the diagnostic capabilities for perihilar biliary strictures, enabling earlier and more accurate interventions.

### 5.3. Indeterminate Biliary Strictures

Indeterminate biliary strictures are those that lack a definitive mass on noninvasive imaging, such as computed tomography (CT) or magnetic resonance cholangiopancreatography (MRCP). Even after ERCP fluoroscopic evaluations using standard brush cytology and forceps biopsy, these strictures cannot be definitively classified as benign or malignant. In evaluating indeterminate biliary strictures, standard ERCP diagnostic methods, including fluoroscopy-guided biopsies with or without brush cytology, remain the initial approach due to their widespread availability and familiarity. However, when standard ERCP techniques are inconclusive, additional intraductal imaging methods, particularly cholangioscopy, can significantly enhance diagnostic accuracy. A meta-analysis of 12 studies, including one randomized controlled trial, demonstrated that adding cholangioscopy to ERCP increased diagnostic yield by 27% compared to ERCP alone, with a sensitivity improvement from 49.99% to 74.00% [67,68,69]. Notably, this increase in diagnostic accuracy was achieved without a significant rise in adverse events, with pancreatitis and cholangitis being the most commonly reported complications (OR 1.46; 95% CI 0.84–2.51).

Recent advancements in diagnostic evaluation have highlighted the benefit of combining multiple modalities. A randomized study comparing ERCP with digital single-operator cholangioscopy (DSOC) to either modality alone demonstrated significant improvements in diagnostic sensitivity. The combined approach achieved a sensitivity of 77.27%, significantly higher than the 44.4% sensitivity observed with either ERCP or DSOC alone (*p* = 0.033). This combination maintained high specificity and positive predictive value, supporting its safety and effectiveness for accurately identifying biliary stricture pathology [70].

Cholangioscopy allows direct visualization of the bile duct, aiding in the differentiation of benign and malignant strictures based on visual characteristics. In cases of indeterminate biliary strictures, where initial ERCP with brushing and biopsy is inconclusive, cholangioscopy can play an important role by providing direct visualization of the bile duct and aiding in targeted biopsy. Malignant lesions often appear as nodular, papillary, or infiltrative masses with irregular mucosa and prominent neovascularization, though sensitivity varies widely (64–95%) depending on the operator’s expertise and technique [71,72]. Single-operator cholangioscopy (SOC) is favored due to its accessibility and ease of use, despite lower image quality compared to dual-operator systems or reusable scopes. Advances such as artificial intelligence (AI) systems trained on SOC images promise enhanced biopsy targeting and reduced inter-observer variability, though further research is necessary to validate AI’s effectiveness in clinical practice [73,74]. Additionally, the Colombian Association of Digestive Endoscopy recommends SOC over traditional ERCP with brushing and/or biopsy for diagnosing indeterminate biliary strictures due to evidence of improved sensitivity and specificity [75]. Table 4 summarizes the primary cholangioscopy methods available, detailing their specific advantages and limitations to guide optimal diagnostic choices in such complex cases. Figure 3 shows the sequence of images illustrates the steps involved in intraductal balloon-guided direct peroral cholangioscopy.

**Table 4:** Comparative overview of cholangioscopy techniques.

Cholangioscopy Method	Description	Advantages	Limitations
Single-Operator Cholangioscopy (SOC)	Operated through duodenoscope by a single endoscopist	High success rate, stable positioning	High cost, steep learning curve
Dual-Operator Cholangioscopy (DOC)	Requires two operators using an ultra-thin reusable endoscope	Better control and maneuverability	High equipment cost, fragile equipment
Direct Cholangioscopy (DC)	Direct access using ultra-slim endoscope	High-definition imaging, potential for virtual chromoendoscopy	Technically challenging, potential severe adverse events

Overview of single-operator, dual-operator, and direct cholangioscopy: [29,30,60,61,65,71,72,77].

The diagnostic landscape for indeterminate biliary strictures (IDBS) continues to evolve, with DSOC emerging as a critical tool that improves accuracy beyond conventional sampling methods. A retrospective study of 35 patients indicated that DSOC-guided biopsy had a technical success rate of 100% and a sample adequacy rate of 92.8%. DSOC led to changes in stricture localization in 21.4% of cases, demonstrating its impact on refining diagnoses. This study also noted strong interobserver agreement (kappa 0.871; 93.1% agreement, *p* < 0.001) using the Monaco classification for visual diagnosis, underscoring DSOC’s role in enhancing diagnostic accuracy and consistency [77]. While cholangioscopy provides valuable visual insights, visual impressions alone are insufficient for definitive oncological decisions. Tissue acquisition remains essential, as the recognition of malignancy-associated patterns aids in biopsy targeting, potentially reducing the need for repeat procedures. One study reported a 31% reduction in the number of procedures required and cost savings of approximately EUR 13,000 per patient when cholangioscopy was used compared to standard ERCP alone [78]. Table 5 gives an overview of different techniques for tissue acquisition in biliary strictures.

The SpyGlass Direct Visualization System (SDVS) is a catheter-based cholangioscopy tool that has shown promising outcomes. An analysis of SDVS-based interventions in 25 patients over three years indicated a procedural success rate of 96%, with successful visualization and biopsy in 96% of cases. Sensitivity, specificity, and accuracy for visual diagnosis were 100%, 83.3%, and 96%, respectively, while biopsy data showed 100% sensitivity, 73% specificity, and 94.4% diagnostic accuracy. SDVS influenced clinical outcomes in over 80% of complex cases, with minimal adverse events, including only one mild case of cholangitis that was conservatively managed [79].

Super minimally invasive peroral cholangioscopy, which utilizes high-resolution imaging and novel visual criteria, has shown efficacy in enhancing diagnostic accuracy. Features such as microvillous projections, irregular and bleeding-prone vessels, and dike-dam-like protuberances have been linked to neoplastic strictures, while scar-like mucosa suggests non-neoplastic origins [80]. These criteria build on earlier diagnostic models, including the Monaco and Mendoza criteria, by incorporating advanced visualization for clearer differentiation between benign and malignant conditions. Integrating these techniques into clinical protocols could significantly improve diagnostic precision and support tailored treatment strategies.

Percutaneous transluminal clamp biopsy (PTCB) is another minimally invasive technique used for evaluating biliary strictures. A single-center study involving 194 cases demonstrated PTCB’s sensitivity of 81.8% and specificity of 100%, with an 18.2% false-negative rate. Non-cholangiocarcinoma strictures were identified as an independent risk factor for false negatives (OR 7.5; 95% CI 1.74–32.6, *p* < 0.01), emphasizing the importance of cautious interpretation and the potential need for additional diagnostics [81].

Alternative imaging techniques, such as intraductal ultrasound (IDUS) and confocal laser endomicroscopy (CLE), are selectively used in complex cases but are not routine. IDUS can identify malignancy markers, including eccentric wall thickening and irregular margins, while CLE provides cellular-level imaging, detecting malignancies via dark collagen bands and thickened vessels [57,58]. Both methods offer higher sensitivity than standard ERCP but are limited by costs and the need for specialized expertise.

IDUS, an advancement in EUS technology, is valuable for its seamless integration during ERCP procedures. However, its shallow penetration depth limits its observational range. Despite this, IDUS provides more accurate differentiation between benign and malignant stenosis and information on tumor characteristics, such as ductal spread and surgical resectability [82]. It is primarily used for bile duct tumor staging.

In conclusion, while standard ERCP methods are generally adequate for initial evaluation, cholangioscopy-guided biopsies provide substantial diagnostic advantages for indeterminate strictures, particularly when visual assessment and tissue sampling are essential for accurate diagnosis. Intraductal imaging methods such as IDUS and CLE offer additional diagnostic support when needed, depending on clinical context and available resources. These advantages underscore the evolving role of advanced imaging and biopsy techniques in addressing the diagnostic limitations of conventional methods.

Despite their utility, indeterminate biliary strictures continue to pose significant challenges due to the inherent limitations of noninvasive imaging modalities. Indeterminate biliary strictures present unique diagnostic challenges due to the inherent limitations of noninvasive imaging modalities. Techniques such as MRI/MRCP and CECT, while essential for initial evaluations, often fall short in distinguishing benign from malignant strictures, particularly in cases where operator expertise and patient-specific factors affect imaging quality [22,25]. Additionally, advanced intraductal imaging tools like confocal laser endomicroscopy (CLE) offer high sensitivity but are underutilized due to their high costs and the need for specialized training [57,58]. As a result, indeterminate cases frequently require repeat procedures to achieve diagnostic clarity, leading to increased patient anxiety and healthcare expenditures.

The future of diagnosing indeterminate biliary strictures lies in the integration of advanced imaging technologies with AI-driven tools. AI-enhanced imaging, such as CNNs applied during cholangioscopy, has shown potential to improve diagnostic yields and reduce inter-observer variability [59]. Moreover, innovative biopsy devices, including novel biliary biopsy cannulae, offer significantly higher malignancy detection rates compared to conventional brushing techniques, minimizing diagnostic uncertainty [83]. AI-based predictive models for malignant biliary strictures are emerging as valuable tools, enabling more accurate risk stratification and guiding clinical decisions [84]. These advancements are poised to transform the diagnostic landscape for indeterminate biliary strictures, reducing the need for invasive procedures while ensuring timely and accurate diagnoses.

## 6. Artificial Intelligence

Artificial intelligence (AI), particularly through deep learning (DL) algorithms such as convolutional neural networks (CNNs), has become a powerful tool in medical imaging. In the diagnosis of biliary strictures and cholangiocarcinoma (CCA), these technologies enhance endoscopic imaging by automating the detection and differentiation of malignant and benign strictures [85,86,87]. Recent studies demonstrate that CNN-based algorithms used with cholangioscopy and endoscopic ultrasound (EUS) significantly improve diagnostic accuracy. For example, CNN-enhanced cholangioscopy has shown impressive diagnostic performance metrics, achieving an accuracy of 94.9%, sensitivity of 94.7%, and specificity of 92.1% [88,89,90,91]. A CNN trained on 84,994 images from digital single-operator cholangioscopy exams demonstrated an accuracy of 82.9%, sensitivity of 83.5%, and specificity of 82.4%, with an AUROC of 0.92, further highlighting AI’s ability to differentiate benign from malignant biliary strictures [92]. Similarly, CNNs integrated with EUS have demonstrated real-time station recognition and bile duct segmentation, significantly improving diagnostic efficiency and providing immediate feedback to the endoscopist [92]. Direct visualization and interpretation of cholangioscopy images using CNNs provide significantly greater accuracy for biliary stricture classification compared to traditional ERCP sampling techniques, with CNN-based video analysis achieving an accuracy of 90.6%, outperforming brush cytology (62.5%) and forceps biopsy (60.9%) [91]. These results underscore the potential of AI to support more precise visual diagnosis and targeted biopsies during cholangioscopy, making it an invaluable adjunct to conventional methods.

Additionally, an AI system using digital single-operator cholangioscopy exhibited outstanding diagnostic accuracy, reliably identifying malignant biliary strictures and associating predictions with key endoscopic features such as nodular masses and abnormal vessels [74]. Integrating AI into practice may also decrease the occurrence of false negatives and aid decision-making in complex cases. In a comparative analysis, a DSOC-AI model achieved diagnostic performance comparable to biopsy-based methods, demonstrating its capability to serve as a reliable tool for identifying malignant biliary strictures, especially in challenging cases [93].

A case report highlighted the real-world utility of the AIWorks-Cholangioscopy system in diagnosing challenging indeterminate biliary strictures, showcasing its ability to complement traditional methods by accurately identifying adenocarcinoma through targeted biopsies [94]. AI-assisted imaging shows promise in improving diagnostic sensitivity and specificity, potentially outperforming traditional ERCP methods, which rely on brush cytology and biopsies with limited sensitivities of 43% and 48%, respectively [95]. The automation and detailed analysis offered by AI can address the complexities of interpreting visual findings, which can be challenging even for experienced endoscopists [96].

Recent findings indicate that AI tools, specifically computer-aided detection (CADe) and computer-aided diagnosis (CADx), enhance sensitivity and accuracy when evaluating biliary strictures. CADe improves sensitivity by prioritizing high-risk cytologic areas for expert review, while CADx autonomously classifies whole slide images (WSIs) with high specificity and accuracy, streamlining the diagnostic process [91].

A recent study employing machine learning algorithms developed a predictive model for diagnosing malignant biliary stricture (MBS). This dual-center retrospective analysis of 398 patients identified key risk factors, including age, stricture location and length, carbohydrate antigen 199 (CA199), total bilirubin (TBil), and the ratio of direct bilirubin to TBil (DBil/TBil). The Random Forest (RF) model emerged as the most effective, achieving an area under the receiver operating characteristic curve of 0.988, significantly outperforming traditional diagnostic methods. The study demonstrated that machine learning-based predictive models can enhance diagnostic precision and guide clinical decisions by accurately identifying high-risk patients, thereby improving patient prognosis [84].

The real-time image processing capabilities of AI, with speeds of 7–15 ms per frame for cholangioscopy and 200–300 ms per frame for EUS, provide immediate feedback to endoscopists. This rapid processing can reduce procedure times and enhance diagnostic efficiency [43,89,91]. For instance, Zhang et al.’s AI system processed images at a remarkable speed of 53 frames per second, qualifying it as a real-time model with low latency. This capability enhances cholangioscopy performance, enabling precise differentiation between benign and malignant biliary strictures [74].

Despite these promising results, several challenges remain for the widespread adoption of AI, including data quality issues, the risk of algorithm overfitting, and the limited availability of extensive and diverse datasets for validation. Future research should focus on building comprehensive data repositories and conducting trials to assess the impact of AI on clinical outcomes, including mortality and cost-effectiveness [86,94]. Additionally, the development of ethical and legal guidelines is essential to ensure AI systems are implemented safely and effectively under clinical oversight.

## 7. Conclusions

The diagnostic evaluation of biliary strictures requires a multifaceted approach that seamlessly integrates noninvasive imaging, advanced endoscopic techniques, and precise tissue sampling. While methods like MRI/MRCP and ERCP [95] provide valuable initial insights, their diagnostic accuracy is often enhanced by complementary tools such as EUS-FNA and intraductal ultrasound (IDUS), which guide more informed treatment strategies. Despite these effective advancements, challenges related to accessibility, operator dependence, and procedural risks highlight the need for continued innovation.

The potential of AI and machine learning to transform diagnostic precision and workflow efficiency is immense, offering real-time analysis and minimizing interpretation variability. Rigorous research and large-scale clinical trials will be essential to validate their impact on patient outcomes and to ensure seamless integration with existing diagnostic practices.

**Figure 4:**
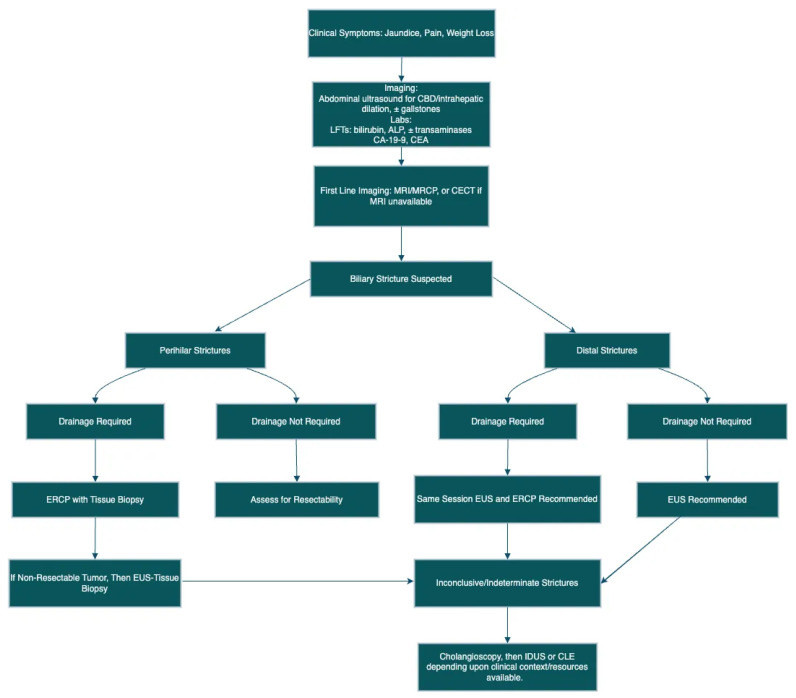
Flowchart depicting the diagnostic algorithm for biliary strictures. Abbreviations expanded: CBD (Common Bile Duct), LFTs (Liver Function Tests), ALP (Alkaline Phosphatase), CA-19-9 (Cancer Antigen 19-9), CEA (Carcinoembryonic Antigen), MRI (Magnetic Resonance Imaging), MRCP (Magnetic Resonance Cholangiopancreatography), CECT (Contrast Enhanced Computed Tomography), ERCP (Endoscopic Retrograde Cholangiopancreatography), EUS (Endoscopic Ultrasound), IDUS (Intraductal Ultrasound), CLE (Confocal Laser Endomicroscopy).

Future efforts should prioritize the development of standardized, cost-effective, and minimally invasive diagnostic strategies that are broadly implementable. Comprehensive training programs to enhance operator skills and equitable access to advanced diagnostic tools are critical. A focus on multidisciplinary collaboration and personalized care will streamline diagnostic pathways, promote timely and accurate diagnoses, and elevate patient care in biliary stricture management. Figure 4 presents a flowchart designed as a diagnostic algorithm for biliary strictures, providing a structured approach to evaluation.

In conclusion, while current advancements provide a strong foundation for diagnosing biliary strictures, sustained research and the thoughtful incorporation of emerging technologies will be crucial for overcoming present limitations and refining diagnostic protocols to improve patient outcomes.

## Figures and Tables

**Table 1:** Main etiologies of biliary strictures.

Malignant Pancreatic Head Carcinoma Gallbladder Carcinoma Cholangiocarcinoma Hepatocellular Carcinoma Ampullary Carcinoma Lymphoma Metastatic Carcinoma
Key Benign Biliary Conditions Choledocholithiasis Mirizzi Syndrome Post cholecystectomy Strictures Sclerosing Cholangitis Choledochal Cyst Chronic Pancreatitis IgG4-mediated cholangitis Sarcoidosis Recurrent pyogenic cholangitis Extrinsic compression by pancreatic fluid collections

**Table 2:** Tumor markers for malignancy in biliary strictures.

Tumor Marker	Sensitivity (%)	Specificity (%)	Diagnostic Utility	Limitations
CA 19-9	74	41.5	Prognosis, recurrence, staging	Elevated in benign conditions like cholangitis
CEA	Variable	Low	Prognosis, malignancy detection	Not specific for pancreato-biliary malignancies

Reference: CA 19-9 and CEA utility and limitations: [11,12,15,17,18,19].

**Table 3:** Diagnostic yield of imaging techniques for biliary strictures.

Imaging Technique	Sensitivity (%)	Specificity (%)	Advantages	Limitations
Ultrasound	31–100	71–97	Cost-effective, accessible, non-invasive	Limited in differentiating stricture origin
MRI/MRCP	81–100	84–100	High-resolution imaging, no radiation	Expensive, requires expertise
CECT	89	96	Fast, good spatial resolution	Radiation exposure, contrast required

Ultrasound, MRI/MRCP, CECT data: [20,22,24,25,26,27].

**Table 5:** Techniques for tissue acquisition in biliary strictures.

Method	Sensitivity (%)	Procedure Type	Strengths	Limitations
ERCP Brush Cytology	40	Endoscopic	Widely available	Low sensitivity
ERCP with Biopsy	60	Endoscopic	Higher sensitivity than brushing	Invasive, risk of complications
EUS-FNA	83	Endoscopic Ultrasound	High sensitivity for distal strictures	Risk of bile leakage, requires experience
Cholangioscopy Biopsy	80	Direct Visualization	High precision, direct visualization	Expensive, requires specialized training

Sensitivity data for ERCP, EUS-FNA, and cholangioscopy: [5,29,37].

## Data Availability

The data used in this review paper are available in online databases.
